# Evaluating the influence of taxation and social security policies on psychological distress: A microsimulation study of the UK during the COVID-19 economic crisis

**DOI:** 10.1016/j.socscimed.2024.116953

**Published:** 2024-05-09

**Authors:** Daniel Kopasker, Patryk Bronka, Rachel M. Thomson, Vladimir Khodygo, Theocharis Kromydas, Petra Meier, Alison Heppenstall, Clare Bambra, Nik Lomax, Peter Craig, Matteo Richiardi, Srinivasa Vittal Katikireddi

**Affiliations:** a MRC/CSO Social and Public Health Sciences Unit, University of Glasgow, UK; b Centre for Microsimulation and Policy Analysis, University of Essex, UK; c Population Health Sciences Institute, Newcastle University, UK; d School of Geography, University of Leeds, UK; e School of Political and Social Sciences, University of Glasgow, UK

**Keywords:** Economic determinants of health, Microsimulation, Social security, Mental health, Policy evaluation

## Abstract

Economic determinants are important for population health, but actionable evidence of how policies can utilise these pathways remains scarce. This study employs a microsimulation framework to evaluate the effects of taxation and social security policies on population mental health. The UK economic crisis caused by the COVID-19 pandemic provides an informative context involving an economic shock accompanied by one of the strongest discretionary fiscal responses amongst OECD countries.

The analytical setup involves a dynamic, stochastic, discrete-time microsimulation model (SimPaths) projecting changes in psychological distress given predicted economic outcomes from a static tax-benefit microsimulation model (UKMOD) based on different policy scenarios. We contrast projections of psychological distress for the working-age population from 2017 to 2025 given the observed policy environment against a counterfactual scenario where pre-crisis policies remained in place. Levels of psychological distress and potential cases of common mental disorders (CMDs) were assessed with the 12-item General Health Questionnaire (GHQ-12).

The UK policy response to the economic crisis is estimated to have prevented a substantial fall (over 12 percentage points, %pt) in the employment rate in 2020 and 2021. In 2020, projected psychological distress increased substantially (CMD prevalence increase >10%pt) under both the observed and the counterfactual policy scenarios. Through economic pathways, the policy response is estimated to have prevented a further 3.4% pt [95%UI 2.8%pt, 4.0%pt] increase in the prevalence of CMDs, approximately 1.2 million cases. Beyond 2021, as employment levels rapidly recovered, psychological distress returned to the pre-pandemic trend. Sustained preventative effects on poverty are estimated, with projected levels 2.1%pt [95%UI 1.8%pt, 2.5%pt] lower in 2025 than in the absence of the observed policy response.

The study shows that policies protecting employment during an economic crisis are effective in preventing short-term mental health losses and have lasting effects on poverty levels. This preventative effect has substantial public health benefits.

## 1 Introduction

The economic circumstances people experience are well-established determinants of mental health (Allen et al., 2014). Employment status has consistently been shown to be particularly influential (Kromydas et al., 2021; Paul and Moser, 2009). Similarly, changes in income, especially when this results in exposure to poverty, have been shown to affect mental health (Thomson et al., 2022a). Tax and social security policies directly influence these economic determinants and could be harnessed to improve health outcomes and reduce inequalities (Simpson et al., 2021). The benefits of influencing health outcomes through such policies are potentially larger than the effects of equivalent healthcare spending (Stuckler et al., 2010). Consideration of health effects from policies targeting primary outcomes outside the healthcare context is a feature of a ‘Health in All Policies’ (HiAP) approach to government (Leeuw, 2017). The HiAP approach has the potential to provide efficient and effective improvements in multiple priority policy areas simultaneously. Such policies could be particularly beneficial during periods of economic crisis. However, few existing models can provide actionable evidence on the health and economic benefits of these policies. This study addresses this evidence gap by means of a microsimulation model that combines tax and social security policies, economic outcomes, and health outcomes to assess the potential of a HiAP approach to influence population mental health.

Microsimulation is a promising method to support evidence-based policymaking when empirical evaluations are either unavailable or may take many years to emerge (Kopasker et al., 2023). Some notable health-focused studies have adopted this method (Kypridemos et al., 2018; Skarda et al., 2021). Complex inter-relationships between determinants, ethical issues with real-world experiments, and other political and practical constraints have restricted evidence of how taxation and social security policies can influence health through economic determinants. Microsimulation can overcome these problems by providing a simplification of the real world that integrates observed data, evidence, and theory to project outcomes from policies that have not necessarily been implemented. In microsimulation, subjects are cycled through a series of processes that each predict a specific outcome variable, based on institutional rules, environmental constraints, and empirically estimated and validated relationships. When individuals are the primary unit of analysis within a microsimulation model, multiple economic determinants of health can interact with other characteristics in large nationally representative samples. Dynamic microsimulation models of the type used in this study repeat the microsimulation process over multiple cycles. This enables projections over longer time horizons and allows for evolution in the characteristics and behaviours of individuals.

The economic crisis in the UK caused by the COVID-19 pandemic provides an informative context in which to study the economic determinants of health since it involved a substantial economic shock (a 27% contraction in gross domestic product between January and June 2020) accompanied by one of the strongest discretionary fiscal responses among OECD countries (HM Treasury, 2023). As with other periods of economic crisis, the macroeconomic shock, principally a fall in labour demand due to pandemic-related work restrictions and related changes in aggregate demand, was experienced at the micro-unit level (individuals and households) through earnings and employment transitions. The observed policy response involved the rapid introduction of innovations across a range of social security policies, plus employment protection not previously used in the UK. This included a temporary uplift in the level of the main social security payment and the introduction of an extensive job retention scheme, amongst other things. Similar policy responses were observed in other countries as governments attempted to limit the impact of the macroeconomic shock through various combinations of increases in social security payments and temporary job retention schemes. Using the SimPaths microsimulation framework (Bronka et al., 2023), we contrast projected levels of psychological distress and cases of common mental disorders (CMDs) in the UK working-age population under the observed policy response to the economic crisis against a counterfactual scenario where only pre-crisis policies remained in place (as has been common during most periods of economic crisis in the UK). Our focus is on the policy response to the economic crisis, rather than the broader public health policies applied during the pandemic. The main hypothesis to be tested is that the policy response limited increases in psychological distress and CMDs during the economic crisis by influencing economic determinants of mental health. Support for this hypothesis will provide initial evidence for the scale and nature of changes required for tax and social security policies to improve population mental health more generally. While the focus is on the UK, the analysis can inform the structure of policy responses to economic crises in other countries with well-developed tax and social security systems.

## 2 Methods

SimPaths is an open-source (https://github.com/centreformicrosimulation/SimPaths) microsimulation model that provides a flexible framework to project life course trajectories across a range of domains, predicting amongst others income, employment, and health outcomes (Bronka et al., 2023). The framework integrates an existing static tax-benefit microsimulation model, UKMOD, which is used to compute tax and social security payments in each simulated period (Richiardi et al., 2021).

UKMOD provides short term, or ‘morning after’, effects on disposable income of (actual or counterfactual) tax and social security policies, including those made by devolved administrations of the UK. Wealth, capital, and indirect taxes are currently not included in UKMOD. UKMOD uses data from the Family Resources Survey (FRS), a household survey that is representative of the UK population.

**Fig. 1:**
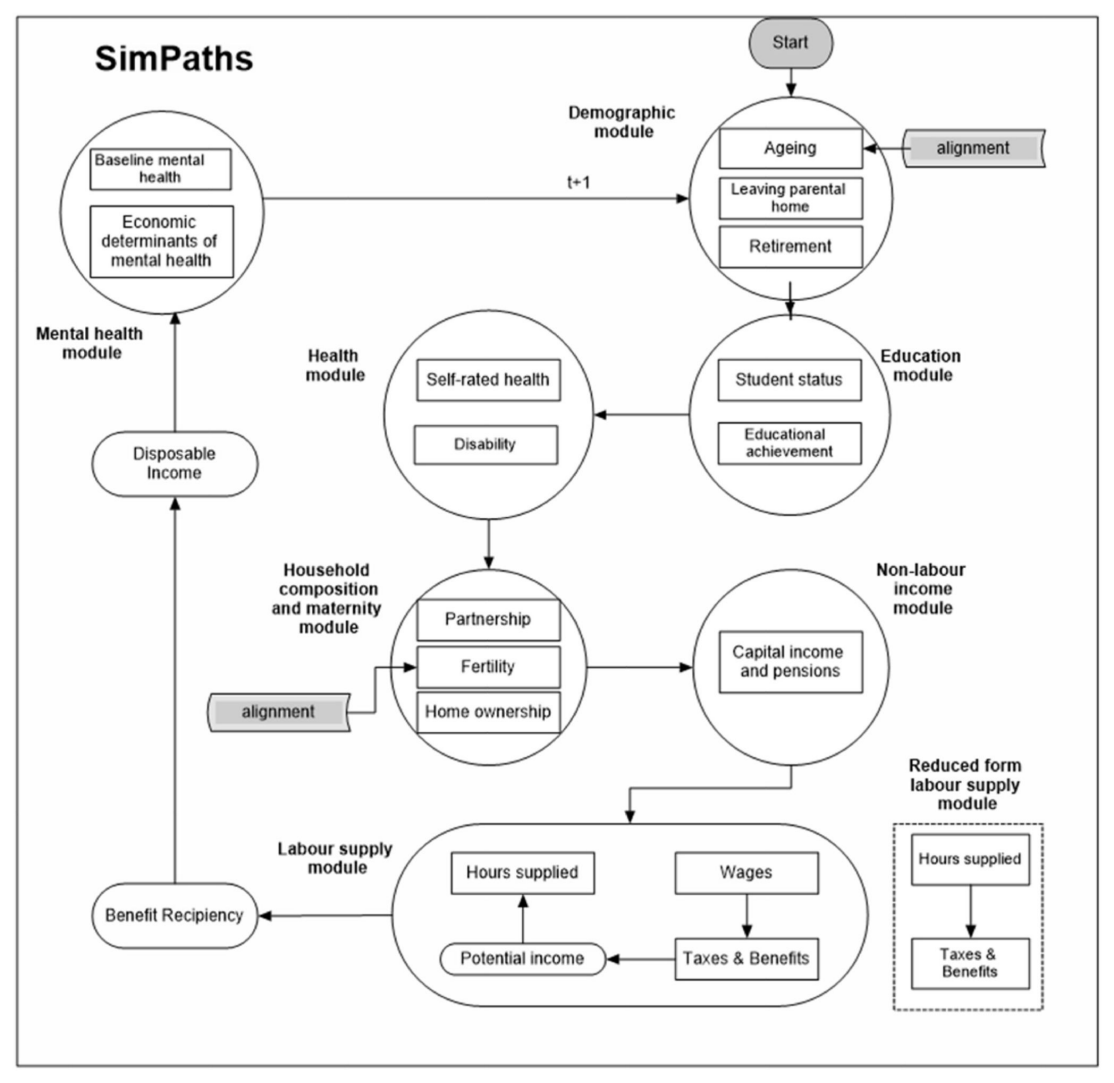
Schematic representation of the SimPaths microsimulation model Notes: Alignment adjusts the simulated population to match official population projections distinguished by gender, age, and geographic region. Reduced form labour supply module used during economic crisis only (2020 and 2021) to account for adverse labour market conditions.

SimPaths estimates employment states and gross incomes (including labour incomes, capital incomes, and pensions) in each simulated year, based on a set of individual determinants (including household structure and prior health). The estimation processes are described later and summarised in Fig. 1. Gross incomes are transformed into disposable income at the household level using UKMOD. Disposable income – and changes thereof – then affect other outcomes in SimPaths, including health outcomes. The model is dynamic in the sense that the characteristics of individuals and households evolve over time based on processes included within the model, and the simulated population changes in line with the demographic processes modelled. This allows changes in outcomes to manifest over multiple periods. Importantly, simulated decisions made by individuals regarding labour supply are influenced by the net income level computed by interaction with UKMOD. This means that individuals in the model react to changes in the fiscal incentives: for example, by increasing or reducing hours worked as a result of changes in taxes and social security payments. The model therefore provides a behavioural link between tax and social security policies and employment outcomes.

The simulated population in SimPaths is drawn from Understanding Society (UKHLS), the main household longitudinal survey in the UK (University of Essex, Institute for Social and Economic Research, 2021a, 2021b). The simulation takes into account weights from Understanding Society and external population projections to ensure the simulated population in SimPaths is representative of the UK population. As the initial population will be smaller than the UK population, a scaling factor is calculated that is constant across individuals and preserves the representativeness of the sample. The scaling factor indicates how many individuals in the UK population are represented by each simulated individual. In the current study, the initial population in the simulation comprises 150,000 individuals, with a constant scaling factor of 446. This approximates a UK population of 66.9 million (150,000 x 446). The sample size was selected to enable analyses of population sub-groups while limiting the time taken to run the computationally demanding elements within the model. Although the whole population is simulated, the results focus on the working-age population (age 25–64) which is a sub-sample of approximately 77,400 individuals. This sub-group is selected due to the causal framework underpinning the analysis being based on this age group and abstracting from employment and income changes resulting from leaving education or entering retirement (Thomson et al., 2022b). The analysis performed in this study covers the period from 2017 to 2025 (other start and end periods can be selected in SimPaths).

The University of Essex Ethics Committee has approved all data collection on Understanding Society main study and innovation panel waves. Ethics approval was granted by the University of Essex Ethics Committee for the COVID-19 web and telephone surveys (ETH1920-1271). The March 2021 web survey was reviewed and ethics approval granted by the NHS Health Research Authority, London – City & East Research Ethics Committee (reference 21/HRA/0644). No additional ethical approval was required for this study.

### 2.1 Policy scenarios

Two policy scenarios are compared. The observed (baseline) policy scenario includes all tax and social security announcements made by the UK and devolved governments up to and including 2021. Importantly, this includes the policy response to the COVID-19 economic crisis. The most notable aspects of this policy response included a £20 per week uplift in Universal Credit (the main social security payment for working-aged people in the UK, which was £73 per week in 2019 for a single person aged 25 or over), and the Coronavirus Job Retention Scheme (CJRS) that was designed to protect employment by providing temporary furlough. The CJRS had a number of iterations during the crisis.

When first introduced the scheme paid 80% of a furloughed employee’s usual wages up to a maximum of £2500. In the counterfactual scenario there was no specific policy response to the economic crisis, such that all tax and social security policies in place or pre-announced by 2019 remained unchanged throughout the pandemic. A more detailed summary of the policy differences between scenarios is included within the supplementary information (Table A7). Contrasting output from the observed and counterfactual policy scenarios provides estimates of the effects on mental health of more generous social security payments and stronger employment protection.

### 2.2 Model structure

A detailed description of all elements of SimPaths is published separately (Bronka et al., 2023), here we provide a summary with a focus on the most important elements for this study. The microsimulation model is structured as a series of modules that update the data in each simulation cycle (as shown in Fig. 1). A simulation cycle is equivalent to one simulated year in this study. Modules are groups of prediction processes for related individual- and household-level state variables (outcomes). The prediction processes are based on regression models estimated on observed data (UKHLS) prior to the simulation commencing. The initial cycle uses observed data from UKHLS. Subsequent cycles use the predicted data from the prior cycle. The entire simulated population passes through each module in each cycle of the simulation. State variables can be an event (or category of an event) occurring, or the level of a continuous variable. For example, two processes are included within the Education module: predictions for student status are based on probit models (one for continuing students, and one for individuals who have previously left education but might consider a period of further training), and educational achievement is predicted based on a multinomial probit model, at the time when the students leave education.

State variables currently considered in the model include time (year), age, sex, region of residence (NUTS1 level), education (three levels), household status (with parents, single, partnered), activity status (student, employed, not employed, retired), potential hourly wage, hours worked, employment earnings, social security payments, capital income, pension income, disposable income, home ownership, self-rated health status (on a 1 to 5 scale), psychological distress (0–36 on a Likert scale; caseness-based measure), long-term sick or disability status (true, false). Individuals are linked to other family members (partner, children, parents) so that their characteristics (including for instance number and age of children, maternal and paternal education, even when not living in the same household) can also be controlled for. The evolution of all state variables is determined within the model, i.e. all state variables are outcomes of some process, in addition to being drivers of other processes.

Each SimPaths cycle (equivalent to one simulated year) begins with an alignment process to ensure the simulated population mirrors the official UK population projections produced by the UK Office for National Statistics in terms of demographic structure (age, sex, and region) and population size. Separate statistical processes, organised in different modules (Demographic, Education, Health, Household composition and fertility, and Non-labour income) are then called in turn, each updating a specific individual-level state variable based on the pre-determined values of the other state variables.

Regression models are deemed sufficient for the microsimulation process if they result in plausible projections of state variables, as determined by an extensive model validation process. Adding complexity or variables to the regression models results in greater computational demands and/or further state variables to be simulated.

The key module for estimating economic outcomes in each simulation cycle is the Labour Supply Module, following a discrete choice random utility specification (Richiardi and He, 2021). The approach considers that labour supply of each partner is jointly decided at the tax unit (usually household) level to maximise the joint utility of the tax unit. Preferences are estimated in the data and depend on income (the more the better) and hours worked (the less the better). State variables affecting preferences (e.g. education, gender, number and age of children) are also considered. This modelling strategy assumes that all outcomes are produced by choices – in other words, there is no involuntary unemployment because not working is a voluntary choice, at the estimated wage rates commanded by the individual characteristics. This might seem an oversimplification, but has the advantage of allowing choices at the extensive (to work or not to work) and the intensive (how much to work) margin to depend on fiscal incentives. For example, people may choose to reduce the hours they work if tax rates rise substantially. This assumption ensures a behavioural link, through labour supply choices, between tax and social security policy interventions and health outcomes. The low level of unemployment in the UK corroborates – to an extent – this approach. Model validation indicates a good fit with the observed data, such that this assumption does not result in unrealistic projections of the employment rate.

The Labour Supply Module begins with a process to estimate potential wages for each individual, using a Heckman-corrected wage equation to control for the fact that in the data wages are observed only for employed individuals (Heckman, 1979). Potential earnings are then calculated for five levels of labour supply of each partner at risk of work (0, 10, 20, 30, or 40 h per week; individuals are not at risk of work if they are students, retired, or long-term sick or disabled), determining up to twenty-five different levels of gross income, for each tax-benefit unit (a tax-benefit unit generally comprises one or two adults, and their dependent children). In each alternative, each simulated tax-benefit unit is then matched to one UKMOD tax-benefit unit with corresponding socio-demographic characteristics (state variables). A gross-to-net income ratio is then imputed, in each alternative, from the UKMOD tax-benefit unit to the simulated household, in order to produce, for each alternative, a measure of disposable income (when gross incomes are below a threshold, disposable income is directly imputed) (van de Ven et al., 2022). This effectively reconstructs the household budget constraint, even if only for the discrete choices considered. Utility is then computed for each tax-benefit unit based on estimated coefficients, adding a random (unobserved) component from an estimated distribution of error terms. Tax-benefit units then choose the level of labour supply of each partner that maximises the joint utility of the tax-benefit unit.

The structural Labour Supply Module is used in most years of the simulation to determine the number of hours each simulated individual works. However, during the main pandemic period (2020 and 2021) additional employment states (furlough and partial furlough) existed, and labour demand declined considerably, making the no involuntary unemployment assumption implausible. In these two years only, a reduced form labour supply module is used, where transition probabilities between the different employment states are directly estimated using in data coming from the Understanding Society COVID-19 study (University of EssexInstitute for Social and Economic Research, 2021,b). Five multinomial logit models are estimated, where the number of categories for the outcome depends on the transitions that are possible given the starting employment state (see Table A6 for all possible transitions). This process provides one prediction equation from each starting employment state to each possible end employment state that involves a transition. For example, one prediction equation is estimated for transitions from employment to non-employment, another for employment to full furlough, and so on. The prediction models control for gender, age, education, and household type. A further model then predicts working hours, if this is required by the predicted employment state. In the counterfactual scenario without the policy response to the economic crisis, the furlough state does not exist, and individuals become more likely to transition to non-employment when labour demand falls. This modelling strategy is agnostic about what motivates the transitions, consistent with the observation that households were heavily constrained during 2020 and 2021, so that labour market transitions were forced upon them, rather than being the outcome of a deliberate choice.

Output from the Labour Supply Module provides projections of all relevant economic outcomes for a single simulation cycle. Projections of psychological distress are then formed in a two-step process based on transitions in economic outcomes between cycles. In the first step, a prediction of psychological distress is made based on observable characteristics but excluding economic outcomes in the current period (see Table A1 for results of the OLS and logit models used). The inclusion of a lagged mental health term in the first step determines the evolution of mental health over time and provides some persistence of health effects from prior economic transitions. In the second step, estimates for each individual in the working-age population are updated with specific direct effects of transitions in income, poverty status, and employment status based on the coefficients from a fixed effects regression. In the version of the model used for this study, these estimates are for short-term effects only. In addition to the effects of economic transitions, in 2020 and 2021 psychological distress increments are applied to all individuals in the simulation for the non-economic effects of the COVID-19 pandemic, since a meta-analysis has shown that the pandemic affected levels of distress regardless of exposure to economic shocks (Bower et al., 2023; Patel et al., 2022).

### 2.3 Model outputs

Psychological distress is measured in UKHLS using the 12-item General Health Questionnaire (GHQ-12), a clinically validated screening tool for psychological distress (Goldberg et al., 1997). Two outcomes are estimated based on this instrument: a Likert score between zero and 36 (with higher scores indicating greater psychological distress), and a binary variable indicating potentially clinically significant CMDs (based on a score of four or more on a zero to 12 scale). CMDs cover depression, anxiety disorders (including generalized anxiety disorder, panic disorder, phobias, social anxiety disorder, obsessive-compulsive disorder, and post-traumatic stress disorder), and combinations (such as mixed anxiety depressive disorder) (National Institute for Health and Care Excellence, 2011). The model estimates both outcomes for all simulated individuals. Individual outcomes are aggregated to give projections for the prevalence of CMDs and the median level of psychological distress in the working-age population. Equivalent results are produced for sub-groups that are important for the study of health inequalities. The model also provides projections for the main pathways of interest, such as the level of poverty or employment resulting from a specific policy scenario.

### 2.4 Key model inputs

With the exceptions of the imputation of gross-to-net income ratio or tax-benefit payments from UKMOD output data (itself derived from the FRS) and related estimates for the random utility labour supply model, all other inputs were estimated using data from UKHLS. An important innovation of SimPaths is to link economic and mental health outcomes by applying increments and decrements to the level of psychological distress for each individual based on their economic transitions in a simulation cycle. These model inputs operationalise the key hypotheses regarding how economic outcomes determine health outcomes. Consequently, the estimation of these effects involved more complex techniques than applied to most other processes within the model. To support variable selection, such that models more precisely estimate the causal direct effects from economic determinants of mental health, a directed acyclic graph (DAG) was drawn which has been peer reviewed and published (Thomson et al., 2022b).

Estimates for the key model inputs are presented in Table 1. Fixed effects regressions (see Tables A4 and A5 for full output) were used to estimate the direct effect of transitions from employed to not-employed (at risk of work), not-employed (at risk of work) to employed, not-employed (at risk of work) to long-term not-employed (at risk of work), non-poverty to poverty, poverty to non-poverty, and poverty to long-term poverty. The same model was used to estimate the effects of changes in the growth rate of equivalised household income and a constant decrement of experiencing any decrease. The growth rate of income, which can be positive or negative, multiplies the coefficient in Table 1. The two income-related coefficients in Table 1 ensure that positive growth rates have positive health effects (and vice versa), larger income changes have larger health effects, and income losses have larger effects than equivalent income gains (Boyce et al., 2013). Household income is used to allow for the effects of partner’s income during economic transitions. Since direct effects are used, the coefficients can be interpreted as the effect without mediation by other economic transitions. Most importantly, this means that the health effect of an employment transition is in addition to any associated income effect, such that they represent the non-pecuniary effects of employment. Effects of economic transitions are based on pre-pandemic data to support interpretations generalisable to other periods of economic crisis. The non-economic effects of the pandemic were estimated using a multilevel mixed-effects generalized linear model, as used in Patel et al. (2022). Where possible, the model inputs were externally validated against estimates from systematic reviews (Paul and Moser, 2009; Thomson et al., 2022a).

### 2.5 Uncertainty, sensitivity, and validation

The main inputs of the model, which result from regressions, are estimates. Consequently, there is uncertainty regarding the estimated coefficients used by the model. To address this source of uncertainty, all regression coefficients are bootstrapped from the corresponding estimated variance-covariance matrixes (variance only used during the second step for mental health outcomes). The bootstrapping process is conducted at the start of each run of the simulation, with a run covering all cycles (years) in a sample period. In effect this means different model inputs are used for each run centred around the point estimates. We run the model 1000 times to provide uncertainty intervals around our central projections. The 25th, 500th, and 975th ranked outcomes are reported.

A detailed description of model validation for SimPaths is published separately (Bronka et al., 2023). In summary, the model was started in 2011 and projections compared to observed data over a ten-year period. Projections were found to provide a close approximation for the observed data. In particular, the mean level of psychological distress predicted by the model was often found to be within one unit (on the 0 to 36 Likert scale) of the observed data for all age-sex groups, especially after the initial model cycles. Additionally, the prevalence of CMDs was found to be within 5%pt of the observed data for the same age-sex groups. Employment and non-employment rates predicted by the model are also comparable to the observed data. Transition rates from employment and non-employment were found to be higher in the simulation than observed data, including external data from the Labour Force Survey five-quarter longitudinal dataset (Office for National Statistics, 2023). However, any overprediction of transition rates will affect both policy scenarios analysed, such that the net effect should largely cancel out when comparing outcomes from two scenarios.

## 3 Results

### 3.1 Economic pathways

**Fig. 2:**
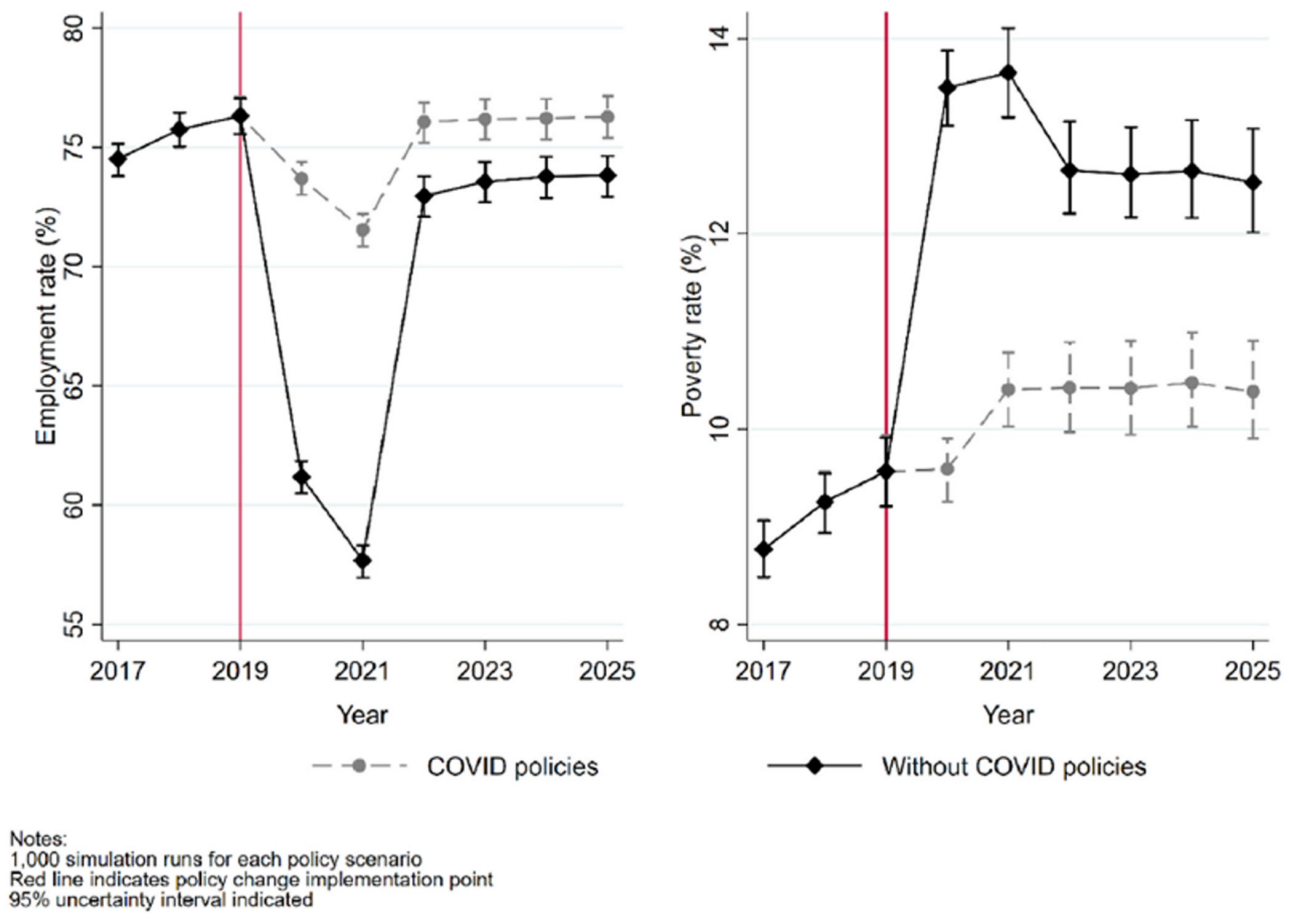
Employment and poverty rates in the UK working-age population from 2017 to 2025 from projections based on the observed policy scenario to the economic crisis and counterfactual policy scenario using pre-crisis policies.

Fig. 2 illustrates trends in two of the economic determinants of health included in the model. Prior to the pandemic, the projected rates of both employment and poverty in the working-age population were stable at around 75% and 9% respectively. The employment rate is estimated to have dropped during 2020 and 2021 under both simulated policy scenarios, but the decrease is projected to have been much larger in the absence of the policy response to the economic crisis. The median difference in the employment rate between the scenarios is 12.5%pt [95% UI 12.0%pt, 13.0%pt] in 2020 and 13.9%pt [95%UI 13.4%pt, 14.4%pt] in 2021. The poverty rate in the counterfactual scenario increases sharply in 2020 to 13.4% [95%UI 13.1%, 13.9%], whereas the rate is stable in the case of the observed policy scenario despite the change in the employment rate. In 2020, the average estimated effect of the observed policy scenario is a 3.9%pt [95%UI 3.6%pt, 4.2%pt] lower rate of poverty compared to the counterfactual scenario, with the equivalent figure for 2021 being 3.2%pt [95%UI 2.9%pt, 3.6%pt].

After the main pandemic crisis period ends in 2022, the estimated employment rates rapidly recover but do not reach the same levels in both policy scenarios. The projected rate of employment within the working-age population in the observed policy scenario is over 75%, while the counterfactual scenario remains under 75%. The median difference in employment rates between policy scenarios in the final simulated period (2025) is 2.5%pt [95%UI 2.0%pt, 2.9%pt], or approximately one million people. For poverty, around 50% of the higher rate in the no-policy intervention (counterfactual) scenario is sustained from 2022 onwards. By the end of the simulation period the poverty rate is 2.1%pt lower [95%UI 1.8%pt, 2.5%pt] due to the observed policy scenario than it was projected to be in the absence of these policies. This represents around 725,000 fewer working-age individuals living in poverty.

### 3.2 Mental health outcomes

**Fig. 3:**
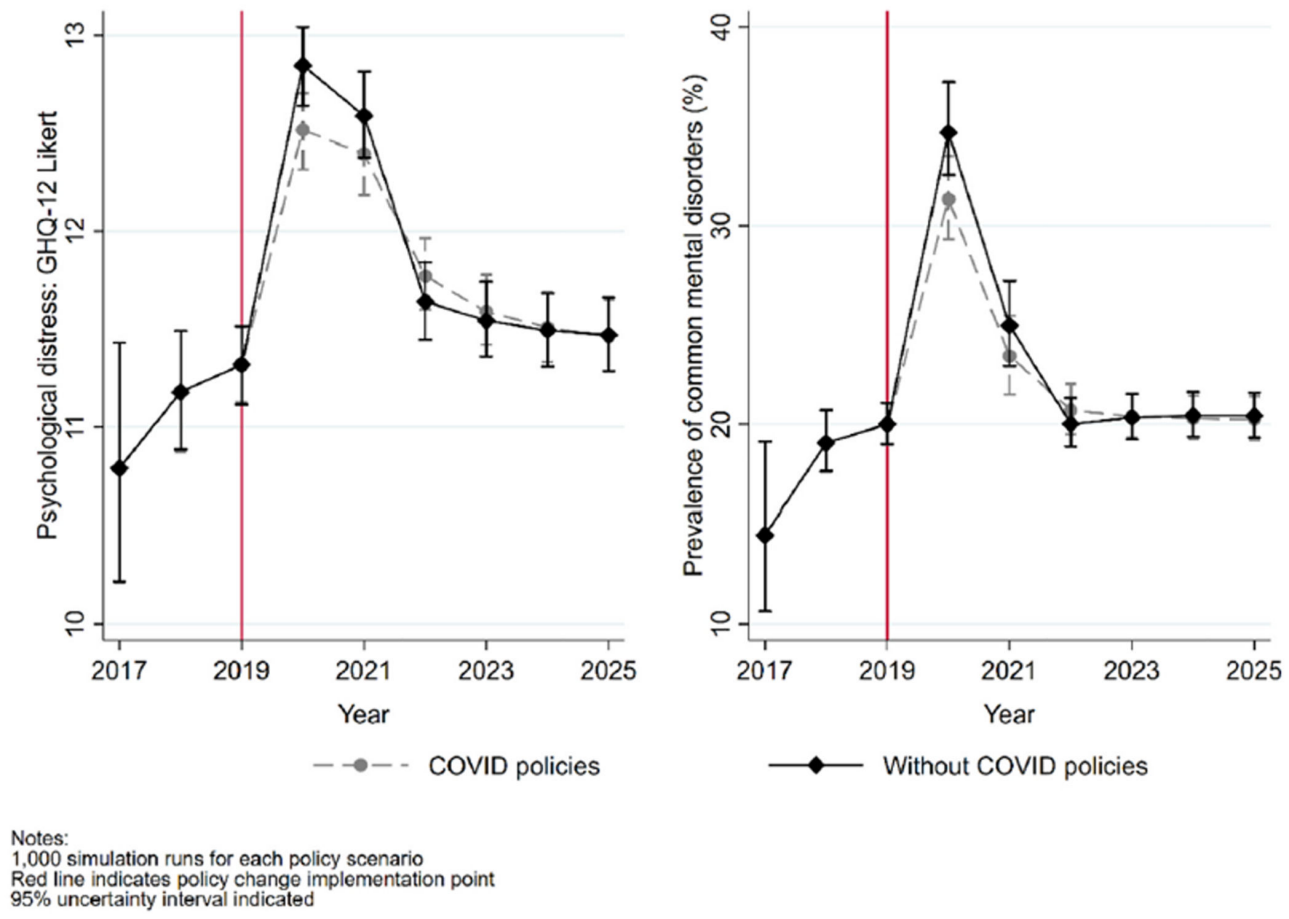
Median levels of psychological distress and prevalence of common mental disorders in the UK working-age population from 2017 to 2025 based on the observed policy scenario to the economic crisis and counterfactual policy scenario using only pre-crisis policies.

Results for the psychological distress outcome variables from the simulation are reported in Fig. 3. Prior to 2019, when implemented policies are identical in the observed and counterfactual scenarios, both the median level of psychological distress and the prevalence of CMDs were estimated to be rising annually. In the three years to 2019, the median level of psychological distress in the working-age population increased from 10.8 [95%UI 10.2, 11.4] to 11.3 [95%UI 11.1, 11.5]. Similarly, the projected prevalence of CMDs also increased from 14.5% [95%UI 10.7%, 19.1%] to 20.0% [95%UI 19.0%, 21.0%].

Although a pre-crisis upward trend in psychological distress is estimated, the effects of the pandemic are substantial and clearly above what would be expected by a continuation of the pre-crisis trend. For the observed policy scenario, the prevalence of CMDs is projected to have increased to 31.3% [95% UI 29.3%, 33.5%] in 2020 and 23.4% [95% UI 21.5%, 25.5%] in 2021. Equivalent projected prevalence for the counterfactual policy scenario is 34.7% [95% UI 32.6%, 37.2%] in 2020 and 24.9% [95% CI 22.9%, 27.2%] in 2021. Using the Likert score measure there was also a substantial increase in estimated median psychological distress to 12.5 [95% CI 12.3, 12.7] in 2020 and 12.4 [95% CI 12.2, 12.6] in 2021 for the observed policy scenario, and 12.8 [95%UI 12.6, 13.0] in 2020 and 12.6 [95%UI 12.4, 12.8] in 2021 for the counterfactual policy scenario.

**Fig. 4:**
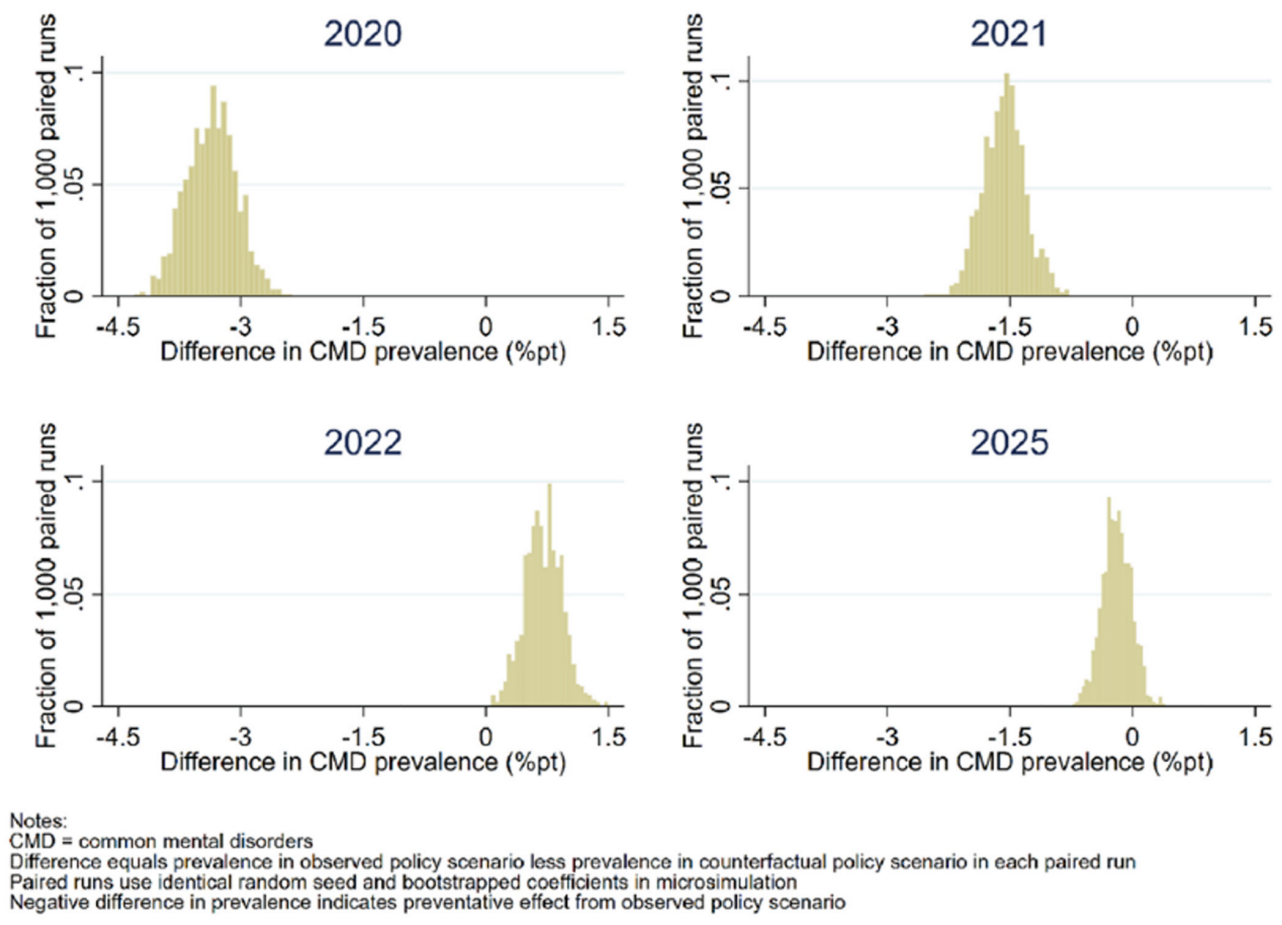
Difference between observed and counterfactual policy scenarios in the prevalence of common mental disorders in selected years using paired simulation runs with the same bootstrapped coefficients.

In all simulated time periods in Fig. 3, there is some crossover in the uncertainty intervals within each policy scenario. Fig. 4 provides histograms showing the difference in projected prevalence of CMDs when estimating the within-year effect from paired runs of the simulation. Paired runs use the same bootstrapped coefficients for both the observed and counterfactual policy scenarios simulated. Negative values indicate a preventative effect within year from the policies introduced in response to the economic crisis. All 1000 estimates of outcomes indicate the policy response restricting cases of CMDs within the UK population in 2020 and 2021. In 2022 this pattern is reversed as the labour market rapidly recovers to pre-crisis levels of labour demand. This enabled individuals who became non-employed at the start of the economic crisis to experience mental health benefits from returning to employment, something that was not possible to the same extent in the observed policy scenario since people were prevented from becoming non-employed by the CJRS. However, it should be noted that the central point of the estimates is smaller in absolute terms than in 2020 and 2021, suggesting that the annual benefits of the observed policy scenario during those years were greater than the benefits achieved by the non-employed returning to employment in 2022. Thereafter, trends in both scenarios are closely matched and plausibly a continuation of the precrisis trends. This pattern of results indicates a benefit from the policy response followed by a rapid return to the pre-crisis trend, a pattern that is also observed for median levels of psychological distress.

### 3.3 Inequalities

One important aspect of the policy response to the economic crisis was the efforts made to protect employment and income. Given the social patterning of these determinants, associated health inequalities could have been impacted. At the start of the pandemic period (2019) the median level of real equivalised net household income is estimated in the model to be £16,503 per year [95%UI £16,267, £16,741], in 2015 prices. Income inequality, as measured by the ratio of the 90th to the 10th percentile of the income distribution, was estimated to be 4.12 [95%UI 4.04, 4.21]. By 2025, median income was £17,291 [95%UI £16,933, £17,564], and income inequality was 4.30 [95%UI 4.13, 4.48] in the observed policy scenario. In the counterfactual policy scenario, the equivalent figures are £16,196 [95%UI £15,854, £16,530] for median income, and 5.19 [95%UI 4.96, 5.41] for income inequality. These estimates indicate that the policy response to the economic crisis increased median household income relative to the 2019 level, and prevented a substantial increase in income inequality that is projected if the policies had not been introduced.

Analyses for health effects from the policy intervention in 2020 by sub-groups important for health inequalities are presented in Table 2 (more detailed results are available in Tables A2 and A3). While it should be noted that the uncertainty intervals for sub-groups often either share some values with the relevant comparator group or include zero, the median values do indicate some potentially important differences: the observed policy scenario had a relatively greater estimated preventative effect in 2020 (and to a lesser extent 2021) for males, households with children, and those with low educational attainment. In each of these cases the preventative effect of the policy response on the prevalence of CMDs is almost two times larger than the relevant comparator group (females, households without children, and high educational attainment). The scale of the differences between groups is estimated to have narrowed in 2021 for all sub-groups except males and females. By 2025 the remaining differences between CMD prevalence amongst sub-groups are small, with the largest difference being a greater preventative effect for households with children. Both high- and low-income sub-groups have estimated policy effects below the overall level, indicating that middle income groups benefited most from the policy response to the economic crisis. However, for the low-income group only, no preventative effect from the policy response was estimated in any period. This suggests that those not reached by the policy response, such as individuals affected by non-employment during the economic crisis, were still more likely to experience economic hardship that resulted in potential cases of CMDs.

## Discussion

Our modelling suggests that income and social security policies introduced in response to the COVID-19 economic crisis in the UK restricted increases in psychological distress and CMDs during the pandemic. The projections indicate that the observed policy response prevented higher levels of psychological distress and CMDs within the UK working-age population in 2020. Although the results seem modest in percentage terms, this represents almost 1.2 million potential cases of CMDs estimated to have been prevented in 2020. Despite this effect not being sustained throughout the sample period, preventing short-term disease burden is a beneficial outcome. Health state utility values for mental health conditions indicate that individuals in England suffering from mixed anxiety depressive disorder (the most common mental disorder in the dataset used) experience a utility decrement of 0.13 compared to individuals with no mental health problems (Roberts et al., 2014). The Green Book published by HM Treasury states that the monetary value of a quality-adjusted life year (QALY) to be used in public policy evaluation is £70,000 (HM Treasury, 2022). Therefore, by preventing a substantial number of additional CMD cases in 2020, the estimated value of the health benefit from the policy response to the economic crisis would have been worth around 161,555 QALYs or £11.31 billion in that year. Estimates of the annual costs associated with common mental disorders, both the direct health service costs and indirect costs from lost employment, also indicate substantial economic benefit from preventing cases. Estimated costs per case are £7964 for anxiety and £9311 for depression (both in 2006 pounds) (McCrone et al., 2008). Although these are historical cost levels, they still provide a good indication of indirect costs and clearly show that preventing 1.2 million cases of CMDs generates significant savings. With prevention being a key principle within population health strategies, this demonstrates how protecting employment and income during times of economic crisis can have substantial benefits.

The projections of a rapid labour market recovery following the main pandemic period are consistent with official data showing unemployment rate was 3.9% pre-crisis (Apr–Jun 2019), this increased during the main crisis period to 4.7% (Apr–Jun 2021), then returned to pre-crisis levels (3.8%) by Apr–Jun 2022 (Office for National Statistics, 2022). Likewise, the model inputs for health effects from employment transitions are broadly consistent with those in a relevant meta-analysis (Paul and Moser, 2009). Consistency with the combination of these two sources of evidence indicates that the SimPaths framework has produced plausible projections.

In this study, we have analysed the combined effect of multiple policies that were introduced in response to the economic crisis. Consequently, we do not identify the effect of individual policies. However, from the model inputs (Table 1), the relative magnitude of the health effects of employment are evidently a strong driver of results from the model, and policies protecting employment will account for most of the estimated difference in health outcomes between the policy scenarios. The CJRS is the most prominent policy analysed which protected employment during the crisis, and research using a static microsimulation model (not including health outcomes) identified this policy as providing the main protective mechanism against income shocks within the policy response (Brewer and Tasseva, 2021; Collado et al., 2020). The results produced are also consistent with emerging evidence from a meta-analysis indicating the CJRS was protective for psychological distress during the pandemic (Jacques et al., 2022). The estimated economic value of the health effects can be compared against the official evaluation of the CJRS, which states that this policy cost £70 billion over the full period it was used (March 2020 to September 2021) (HM Treasury, 2023). The official evaluation concluded that the CJRS offered value for money, but did not quantify the indirect health benefits of protecting employment during an economic crisis. The indirect health benefits identified in this study add further support to the conclusion that the CJRS in particular, which preserved employment in addition to protecting incomes, offered value for money and demonstrates the benefits of a HiAP approach to government (HM Treasury, 2023).

To be noted, the CJRS was the first time a furlough scheme has been used in the UK. However, other countries have regularly used short-working schemes during periods of recession to protect employment levels (Balleer et al., 2016; Hijzen and Martin, 2013), and it is likely that benefits to population mental health resulted indirectly from such policies. This is consistent with evidence that employment is one of the main economic determinants of mental health (Kromydas et al., 2021). It is notable that the short-term employment protection simulated using SimPaths resulted in a sustained higher level of employment beyond the main crisis period compared to the counterfactual scenario where the additional employment protection was absent. This is likely to result in valuable economic benefits from the policy, in addition to the short-term health benefits valued above.

Projections of poverty prevalence from the microsimulation could also inform policymaking. The policy response is estimated to have a sustained benefit in terms of preventing exposure to poverty. Reducing poverty levels is often a stated policy goal, particularly within the devolved governments of the UK. The projections indicate approximately 0.73 million fewer working-age adults living in poverty in 2025. Furthermore, the observed policy scenario was estimated to have prevented a substantial increase in income inequality, another long-term policy goal that is particularly difficult to address.

Although health benefits from the observed policy scenario were indicated by the microsimulation, these were relatively modest in comparison to the employment effects and were limited to the short term and the one aspect of health examined. Due to the rapid labour market recovery, some movement back towards parity between the two policy scenarios was expected. However, given the lasting projected effects on poverty, we would expect around 45,000 fewer CMD cases (based on existing evidence) in the observed policy scenario compared to the counterfactual (Kromydas et al., 2021). This may indicate that the simulation, particularly when run 1000 times using different coefficient estimates, is not sufficiently sensitive to identify this relatively small effect.

One observed feature of the pandemic-induced economic crisis in the UK was a rise in the rate of economic inactivity. This increase is partly due to a rise in the level of long-term sickness, with long COVID or delayed medical treatment contributing to this. A process predicting long-term sickness is included within SimPaths, and simulated individuals within this category are considered to be not at risk of work. However, this process was estimated using pre-pandemic period data and factors influencing this outcome may have changed. As a result, it is possible that the model underestimates the number of long-term sick individuals incurring the mental health decrement of transitions to non-employment. Similarly, the process determining retirement decisions may have changed with some people choosing to retire earlier due to the pandemic. It will be possible to address this limitation of the model as data emerges in the future that enables the retirement process to be updated.

One challenge of counterfactual policy analysis is that certain processes may not be consistent in both arms of the model. For example, transitions between employment states in the absence of a policy response to the economic crisis can never be observed and rates may differ from those that are observable. However, the singularity of the event makes it hard to estimate counterfactual transitions, and strongly supports the use of scenario analysis. The severity of lockdown measures makes it likely that without the furlough scheme most affected people would have transitioned to unemployment, our assumed counterfactual scenario.

Further development, including expanding the range of countries and health outcomes modelled, is planned in line with the published protocol for the Health Equity and its Economic Determinants project (Katikireddi et al., 2022). This will include incorporating the cumulative effects of past exposures and allowing health outcomes to enter more processes in subsequent periods. This will enable virtuous, or otherwise, circles between health and economic outcomes. Psychological distress is likely to influence subsequent economic outcomes either directly or indirectly. One example of an indirect influence could be through partnership status with increased psychological distress contributing to partnership dissolution. Following the dissolution of a partnership the individuals would be more limited in the level of labour they can supply, such that their economic outcomes change as the effects of psychological distress accumulate over time across non-economic outcome. The Sim-Paths framework can be readily developed to capture such changes within the projections from the model.

## 5 Conclusion

Policies protecting the income and employment outcomes of individuals and households during periods of economic crisis can have important mental health benefits. The policy response in the UK to the COVID-19 economic crisis has provided an example of the benefits of a HiAP approach to government. The benefits of this policy response can inform the development of tax and social security to be implemented during future periods of economic crisis.

Microsimulation has the potential to overcome barriers that have limited the evidence base for effectively applying a HiAP approach to government across a range of policy areas (Kopasker et al., 2023). The methodological innovation of constructing a microsimulation model to investigate the economic determinants of health creates opportunities to examine the hypothetical use of policies, such as job retention schemes during periods of economic recession, while providing actionable evidence.

It is likely that the effects of economic determinants of mental health manifest over multiple periods and through complex pathways. At this stage, it is unclear if improvements in trends for population health outcomes can be achieved most effectively through immediate policy shifts or using policies implemented (potentially incrementally) over many years. Despite applying a relatively short-term model, this study has shown how tax and social security policies protecting employment and income during times of economic crisis will also influence mental health outcomes. By understanding and utilising these pathways a HiAP approach to government could restrict the societal burden of disease while contributing to other longstanding policy goals.

## Supplementary Material

Supplementary data to this article can be found online at https://doi.org/10.1016/j.socscimed.2024.116953.

Appendix

## Figures and Tables

**Table 1:** Key model inputs for the effects on psychological distress of economic transitions and non-economic pandemic exposure.

	Males Likert	Females Likert	Males Caseness	Females Caseness
Employed to not-employed (at risk of work)	2.74 (0.18)	1.67 (0.14)	1.46 (0.10)	0.76 (0.07)
Not-employed (at risk of work) to employed	− 2.74 (0.19)	− 2.12 (0.15)	−1.26 (0.12)	− 0.93 (0.08)
Not-employed (at risk of work) to long-term not-employed (at risk of work)	− 0.32 (0.22)	− 0.73 (0.14)	− 0.16 (0.12)	− 0.43 (0.08)
Non-poverty to poverty	0.29 (0.10)	0.29 (0.09)	0.03 (0.08)	0.16 (0.06)
Poverty to non-poverty	− 0.33 (0.15)	− 0.12 (0.13)	− 0.10 (0.11)	0.01 (0.08)
Poverty to long-term poverty	0.07 (0.16)	0.07 (0.14)	0.04 (0.11)	0.02 (0.09)
Change in growth rate of household income	− 0.01 (0.04)	− 0.12 (0.05)	− 0.05 (0.03)	− 0.08 (0.03)
Household income decrease	0.10 (0.04)	0.07 (0.04)	0.06 (0.03)	0.01 (0.03)
Pandemic 2020 non-economic effect	0.83 (0.08)	1.41 (0.08)	0.63 (0.08)	0.83 (0.05)
Pandemic 2021 non-economic effect	0.50 (0.08)	0.37 (0.08)	0.08 (0.08)	− 0.04 (0.05)

Notes: Standard error in parentheses.

Individuals are considered to be at risk of work unless they are students, retired, or long-term sick or disabled. Employed includes the self-employed. Psychological distress measured by the 12-item General Health Questionnaire. Likert score ranges from zero to 36 with higher values indicating greater psychological distress. Caseness is a binary variable indicating potentially clinically significant common mental disorders. Effects for caseness are expressed as change in log odds.

**Table 2:** Effect of policy response to the economic crisis on mental health outcomes of sub-groups in 2020.

Year:2020	Psychological distress: GHQ-12 Likert	Prevalence of common mental disorders (%)
Overall	− 0.328 [−0.397,−0.264]	− 3.4 [−4.0,−2.8]
Males	− 0.402 [−0.511,−0.299]	− 4.4 [−5.3,−3.4]
Females	− 0.249 [−0.347,−0.159]	− 2.4 [−3.2,−1.6]
Households with children	− 0.429 [−0.539,−0.322]	− 4.5 [−5.4,−3.6]
Households without children	− 0.268 [−0.348,−0.188]	− 2.7 [−3.3,−2.0]
High education	− 0.232 [−0.337,−0.130]	− 2.4 [−3.1,−1.6]
Low education	− 0.427 [−0.618,−0.190]	− 4.2 [−5.8,−2.7]
Lowest income quintile	0.133 [−0.050,0.303]	−1.0 [−2.4,0.3]
Highest income quintile	− 0.095 [−0.219,0.022]	−1.1 [−2.0,−0.3]

95% uncertainty interval in brackets.

Effect of policy response is outcome from the observed policy scenario less outcome from the counterfactual policy scenario in paired runs of the model. Negative values indicate preventative effect from the observed policy scenario.

## Data Availability

Code available on GitHub: https://github.com/centreformicrosimulation/SimPaths. Data is available from the UK Data Service.
