# Supplementary material for: Evaluating the influence of taxation and social security policies on psychological distress: A microsimulation study of the UK during the COVID-19 economic crisis
Source: Soc Sci Med. Author manuscript; Available in PMC 2025 Apr 11. (PMC7617584; doi:10.1016/j.socscimed.2024.116953)
Supplement: Appendix [file EMS204253-supplement-Appendix.pdf]

Table A1. Prediction model for psychological distress (GHQ-12 Likert score and caseness)

Outcome: GHQ-12	Likert: OLS	Caseness: logit
Benefit income dummy	0.141^{***}	0.041^{*}
	[0.071,0.211]	[-0.006,0.088]
Homeowner dummy	-0.294^{***}	-0.153^{***}
	[-0.380,-0.209]	[-0.201,-0.104]
Single and never married	0.038	0.047
	[-0.073,0.149]	[-0.017,0.110]
Previously partnered	0.001	0.048
	[-0.094,0.096]	[-0.012,0.107]
Lagged number of dependent children	0.022	0.056^{**}
	[-0.070,0.113]	[0.002,0.109]
Lagged self-rated health	-0.581^{***}	-0.354^{***}
	[-0.616,-0.547]	[-0.375,-0.333]
2nd equivalised HH income quintile	-0.021	-0.059^{**}
	[-0.122,0.080]	[-0.116,-0.001]
Middle equivalised HH income quintile	-0.076	-0.057^{*}
	[-0.180,0.028]	[-0.117,0.003]
4th equivalised HH income quintile	-0.113^{**}	-0.116^{***}
	[-0.221,-0.005]	[-0.180,-0.051]
Highest equivalised HH income quintile	-0.137^{**}	-0.109^{***}
	[-0.245,-0.029]	[-0.178,-0.041]
Lagged long-term sick or disabled dummy	1.936^{***}	0.570^{***}
	[1.660,2.213]	[0.471,0.670]
Male dummy	-0.555^{***}	-0.321^{***}
	[-0.619,-0.490]	[-0.364,-0.278]
Age	0.018^{***}	-0.001
	[0.007,0.030]	[-0.009,0.006]
Age squared	-0.000^{***}	-0.000^{***}
	[-0.000,-0.000]	[-0.000,-0.000]
Education: Medium	0.022	-0.048^{*}
	[-0.053,0.097]	[-0.099,0.004]
Education: Low	0.026	-0.043
	[-0.077,0.130]	[-0.113,0.026]
Lagged GHQ-12: Likert	0.489^{***}	
	[0.480,0.499]	
Lagged GHQ-12: caseness		1.671^{***}
		[1.624,1.719]
Year minus 2000	0.020^{***}	0.003
	[0.009,0.030]	[-0.004,0.009]
Region dummies	Yes	Yes
Observations	139954	139954

Standard errors were clustered at the individual level.

95% confidence intervals in brackets * $p < 0.10$, ** $p < 0.05$, *** $p < 0.01$

Table A2. Median psychological distress (GHQ-12 Likert score) and policy effect by post-intervention year and sub-group

	Observed policy scenario	Counterfactual policy scenario	Effect of policy response
Overall			
2020	12.498 [12.314,12.705]	12.847 [12.641,13.043]	-0.328 [-0.397,-0.264]
2021	12.405 [12.184,12.609]	12.589 [12.375,12.816]	-0.199 [-0.268,-0.131]
2025	11.469 [11.281,11.648]	11.469 [11.286,11.661]	-0.007 [-0.068,0.050]
Males			
2020	11.861 [11.564,12.084]	12.23 [11.955,12.499]	-0.402 [-0.511,-0.299]
2021	11.849 [11.607,12.161]	12.156 [11.851,12.454]	-0.268 [-0.380,-0.152]
2025	10.910 [10.766,11.194]	10.99 [10.764,11.218]	-0.013 [-0.106,0.081]
Females			
2020	13.212 [12.943,13.445]	13.455 [13.196,13.709]	-0.249 [-0.347,-0.159]
2021	12.935 [12.621,13.135]	13.022 [12.744,13.284]	-0.131 [-0.228,-0.032]
2025	11.990 [11.739,12.168]	11.941 [11.737,12.170]	0.002 [-0.086,0.090]
Households with children			
2020	12.587 [12.238,12.797]	12.952 [12.663,13.226]	-0.429 [-0.539,-0.322]
2021	12.509 [12.123,12.727]	12.629 [12.310,12.940]	-0.213 [-0.335,-0.099]
2025	11.475 [11.150,11.750]	11.459 [11.166,11.767]	-0.013 [-0.116,0.088]
Households without children			
2020	12.465 [12.313,12.706]	12.786 [12.578,12.984]	-0.268 [-0.348,-0.188]
2021	12.409 [12.165,12.587]	12.562 [12.357,12.797]	-0.191 [-0.271,-0.108]
2025	11.451 [11.288,11.655]	11.475 [11.280,11.655]	-0.002 [-0.075,0.081]
High education			
2020	12.096 [11.919,12.377]	12.388 [12.164,12.612]	-0.232 [-0.337,-0.130]
2021	12.097 [11.830,12.290]	12.210 [11.989,12.449]	-0.159 [-0.258,-0.056]
2025	11.209 [11.020,11.437]	11.226 [11.012,11.440]	0.002 [-0.093,0.105]
Low education			
2020	13.454 [13.126,13.717]	13.834 [13.54,14.119]	-0.427 [-0.618,-0.190]
2021	12.892 [12.855,13.494]	13.38 [13.078,13.726]	-0.230 [-0.452,0.001]
2025	12.112 [11.691,12.297]	12.024 [11.73,12.33]	-0.032 [-0.274,0.211]
Lowest income quintile			
2020	13.716 [13.451,14.044]	13.598 [13.347,13.829]	0.133 [-0.050,0.303]
2021	13.324 [12.991,13.595]	13.155 [12.894,13.441]	0.139 [-0.031,0.296]
2025	13.604 [13.015,13.637]	13.229 [12.911,13.546]	0.092 [-0.128,0.295]
Highest income quintile			
2020	11.812 [11.711,12.117]	12.018 [11.798,12.214]	-0.095 [-0.219,0.022]
2021	11.790 [11.620,12.037]	11.911 [11.685,12.130]	-0.079 [-0.213,0.040]
2025	10.839 [10.708,11.052]	10.869 [10.696,11.036]	0.017 [-0.098,0.130]

95% uncertainty interval in brackets

Effect of policy response is outcome from the observed policy scenario less outcome from the counterfactual policy scenario in paired runs of the model. Negative values indicate preventative effect from the observed policy scenario.

Table A3. Proportion of potentially clinically significant common mental disorders and policy effect by post-intervention year and by sub-group

	Observed policy scenario	Counterfactual policy scenario	Effect of policy response
Overall			
2020	0.312 [0.293,0.335]	0.347 [0.326,0.372]	-0.034 [-0.040,-0.028]
2021	0.236 [0.215,0.255]	0.250 [0.229,0.272]	-0.016 [-0.021,-0.01]
2025	0.205 [0.192,0.214]	0.204 [0.193,0.216]	-0.002 [-0.006,0.001]
Males			
2020	0.268 [0.235,0.297]	0.309 [0.274,0.345]	-0.044 [-0.053,-0.034]
2021	0.206 [0.182,0.239]	0.232 [0.201,0.263]	-0.022 [-0.031,-0.014]
2025	0.179 [0.166,0.191]	0.181 [0.168,0.195]	-0.003 [-0.008,0.002]
Females			
2020	0.359 [0.336,0.388]	0.385 [0.359,0.414]	-0.024 [-0.032,-0.016]
2021	0.263 [0.234,0.282]	0.267 [0.242,0.293]	-0.009 [-0.016,-0.003]
2025	0.225 [0.213,0.241]	0.228 [0.213,0.243]	-0.001 [-0.006,0.004]
Households with children			
2020	0.322 [0.298,0.351]	0.369 [0.342,0.398]	-0.045 [-0.054,-0.036]
2021	0.252 [0.220,0.271]	0.264 [0.237,0.291]	-0.018 [-0.026,-0.010]
2025	0.202 [0.189,0.226]	0.212 [0.193,0.232]	-0.005 [-0.011,0.001]
Households without children			
2020	0.306 [0.285,0.329]	0.334 [0.311,0.359]	-0.027 [-0.033,-0.020]
2021	0.221 [0.209,0.249]	0.241 [0.221,0.264]	-0.014 [-0.020,-0.008]
2025	0.199 [0.190,0.211]	0.200 [0.189,0.212]	0.000 [-0.005,0.004]
High education			
2020	0.301 [0.276,0.320]	0.321 [0.298,0.346]	-0.024 [-0.031,-0.016]
2021	0.216 [0.201,0.241]	0.234 [0.212,0.257]	-0.013 [-0.020,-0.006]
2025	0.194 [0.183,0.208]	0.197 [0.185,0.209]	-0.001 [-0.007,0.005]
Low education			
2020	0.346 [0.336,0.392]	0.405 [0.377,0.437]	-0.042 [-0.058,-0.027]
2021	0.270 [0.245,0.299]	0.289 [0.260,0.317]	-0.016 [-0.031,-0.002]
2025	0.228 [0.205,0.246]	0.228 [0.209,0.250]	-0.003 [-0.019,0.012]
Lowest income quintile			
2020	0.402 [0.364,0.422]	0.403 [0.376,0.433]	-0.010 [-0.024,0.003]
2021	0.280 [0.266,0.318]	0.290 [0.265,0.317]	0.002 [-0.010,0.013]
2025	0.327 [0.316,0.364]	0.336 [0.312,0.361]	0.003 [-0.012,0.019]
Highest income quintile			
2020	0.272 [0.250,0.289]	0.280 [0.259,0.303]	-0.011 [-0.02,-0.003]
2021	0.204 [0.179,0.215]	0.204 [0.184,0.223]	-0.008 [-0.015,0.000]
2025	0.157 [0.153,0.172]	0.162 [0.154,0.172]	0.000 [-0.007,0.006]

95% uncertainty interval in brackets.

Effect of policy response is outcome from the observed policy scenario less outcome from the counterfactual policy scenario in paired runs of the model. Negative values indicate preventative effect from the observed policy scenario.

Table A4. Fixed effects regression for effect of economic transitions on level of psychological distress

	Males GHQ-12 Likert	Females GHQ-12 Likert
Employment transitions (base: not employed - at risk of work)		
Continuously employed	-2.74***	-1.67***
	[-3.08,-2.39]	[-1.93,-1.40]
Newly employed	-2.74***	-2.12***
	[-3.12,-2.36]	[-2.41,-1.84]
Long-term not employed (at risk of work)	-0.32	-0.73***
	[-0.76,0.11]	[-1.01,-0.44]
Growth rate of real equivalised household income	-0.01	-0.12**
	[-0.10,0.08]	[-0.21,-0.02]
Decreased income dummy	0.10***	0.07*
	[0.03,0.17]	[-0.00,0.15]
Poverty transitions (base: poverty)		
Continuous non-poverty	-0.29***	-0.29***
	[-0.49,-0.09]	[-0.47,-0.11]
Newly non-poverty	-0.33**	-0.12
	[-0.62,-0.05]	[-0.37,0.13]
Long-term poverty	0.07	0.07
	[-0.25,0.39]	[-0.20,0.34]
Benefit income dummy	0.01	0.06
	[-0.09,0.12]	[-0.06,0.17]
Homeowner dummy	0.02	0.17
	[-0.20,0.24]	[-0.07,0.40]
Single and never married	-0.27*	-0.28**
	[-0.56,0.02]	[-0.55,-0.00]
Previously partnered	-0.49***	-0.63***
	[-0.82,-0.16]	[-0.91,-0.36]
Lagged number of dependent children	0.03	0.13**
	[-0.10,0.17]	[0.01,0.25]
Lagged self-rated health	-0.07**	-0.04
	[-0.13,-0.01]	[-0.10,0.02]
2nd equivalised HH income quintile	-0.12	0.01
	[-0.35,0.11]	[-0.19,0.21]
Middle equivalised HH income quintile	-0.09	-0.07
	[-0.33,0.16]	[-0.28,0.15]
4th equivalised HH income quintile	-0.12	0.00
	[-0.38,0.14]	[-0.23,0.24]
Highest equivalised HH income quintile	-0.13	0.04
	[-0.41,0.15]	[-0.22,0.30]
Lagged GHQ-12: Likert	-0.03***	-0.03***
	[-0.05,-0.02]	[-0.04,-0.02]
Age	0.33***	0.47***
	[0.18,0.48]	[0.33,0.61]
Age squared	-0.00***	-0.00***
	[-0.00,-0.00]	[-0.00,-0.00]
Education: Medium	-0.15	0.04
	[-0.88,0.58]	[-0.64,0.72]
Education: Low	0.09	0.64
	[-0.93,1.12]	[-0.40,1.67]
Year minus 2000	-0.03	-0.13**
	[-0.15,0.09]	[-0.25,-0.02]
Region dummies	Yes	Yes
Observations	70550	92170

95% confidence intervals in brackets   * $p < 0.10$, ** $p < 0.05$, *** $p < 0.01$

Table A5. Fixed effects regression estimating effect of economic transitions on psychological distress caseness

	Males	Females
	GHQ caseness (over 4)	GHQ caseness (over 4)
Employment transitions (base: not employed - at risk of work)		
Continuously employed	-1.46***	-0.76***
	[-1.65,-1.27]	[-0.90,-0.63]
Newly employed	-1.26***	-0.93***
	[-1.49,-1.03]	[-1.08,-0.77]
Long-term not employed (at risk of work)	-0.16	-0.43***
	[-0.40,0.07]	[-0.58,-0.28]
Growth rate of real equivalised household income	-0.05*	-0.08**
	[-0.11,0.01]	[-0.14,-0.02]
Decreased income dummy	0.06*	0.01
	[-0.01,0.12]	[-0.05,0.06]
Poverty transitions (base: poverty)		
Continuous non-poverty	-0.03	-0.16***
	[-0.18,0.11]	[-0.28,-0.05]
Newly non-poverty	-0.10	0.01
	[-0.32,0.12]	[-0.16,0.17]
Long-term poverty	0.04	0.02
	[-0.18,0.27]	[-0.14,0.19]
Benefit income dummy	0.05	0.03
	[-0.05,0.16]	[-0.05,0.11]
Homeowner dummy	-0.07	0.05
	[-0.25,0.11]	[-0.10,0.20]
Single and never married	-0.11	-0.14*
	[-0.32,0.10]	[-0.30,0.02]
Previously partnered	-0.19*	-0.39***
	[-0.42,0.03]	[-0.55,-0.24]
Lagged number of dependent children	0.05	0.02
	[-0.07,0.16]	[-0.06,0.10]
Lagged self-rated health	-0.07***	-0.04**
	[-0.12,-0.03]	[-0.07,-0.00]
2nd equivalised HH income quintile	-0.09	0.01
	[-0.27,0.09]	[-0.13,0.14]
Middle equivalised HH income quintile	-0.08	0.04
	[-0.27,0.12]	[-0.11,0.18]
4th equivalised HH income quintile	-0.08	0.05
	[-0.29,0.13]	[-0.11,0.21]
Highest equivalised HH income quintile	-0.07	0.11
	[-0.30,0.16]	[-0.07,0.28]
Lagged GHQ-12: caseness	-0.45***	-0.42***
	[-0.51,-0.38]	[-0.46,-0.37]
Age	0.11*	0.22***
	[-0.02,0.24]	[0.13,0.32]
Age squared	-0.00***	-0.00***
	[-0.00,-0.00]	[-0.00,-0.00]
Education: Medium	0.01	-0.03
	[-0.66,0.68]	[-0.42,0.36]
Education: Low	0.05	0.29
	[-0.80,0.89]	[-0.37,0.96]
Year minus 2000	0.01	-0.10**
	[-0.09,0.12]	[-0.18,-0.02]
Region dummies	Yes	Yes
Observations	27652	46133

95% confidence intervals in brackets * $p < 0.10$, ** $p < 0.05$, *** $p < 0.01$

Table A6. Possible transitions between employment states modelled by the reduced-form COVID-19 labour supply module

From/To	Employed	Self-employed	Full furlough	Partial furlough	Not employed
Employed	Y - some changes*<br>Y - no changes	Y*	Y	Y**	Y
Self-employed	Y*	Y*			Y
Full furlough	Y*	Y*	Y	Y**	Y
Partial furlough	Y*	Y*	Y	Y**	Y
Not employed	Y*	Y*			Y

Notes:
Y indicates a transition is possible
* hours of work are predicted in an additional estimation process
** hours of work specific to partial furlough are predicted in an additional estimation process
Full furlough and partial furlough are only used in the observed policy scenario where policies are introduced in response to the economic crisis

Table A7. Summary of policy differences between observed and counterfactual scenarios

Policy	Value (£s per time period) #y – year, #m – month, #w - week	
	Observed With COVID policies	Counterfactual Without COVID policies
Working Tax Credit: Basic element	3040#y	1995#y
Housing Benefit disregards: 16/30 hours earnings disregard	37.1#w	17.1#w
Universal Credit: standard allowances: Single or lone parent 16-17	342.72#m	256.05#m
Universal Credit: standard allowances: Single 18-24	342.72#m	256.05#m
Universal Credit: standard allowances: Single 25 or over	409.89#m	323.22#m
Universal Credit: standard allowances: Lone parent 18 or over	409.89#m	323.22#m
Universal Credit: standard allowances: Couple both under 18	488.59#m	401.92#m
Universal Credit: standard allowances: Couple both over 18 (18-24)	488.59#m	401.92#m
Universal Credit: standard allowances: Couple one or both 25 or over	594.04#m	507.37#m
Coronavirus Job Retention Scheme: earnings replacement rate	0.8	n/a
Coronavirus Job Retention Scheme: maximum payment to furloughed workers	2500#m	n/a
Coronavirus Job Retention Scheme: earnings replacement rate in September 2020 and July 2021	0.7	n/a
Coronavirus Job Retention Scheme: maximum payment to furloughed workers in September 2020 and July 2021	2187.5#m	n/a
Coronavirus Job Retention Scheme: earnings replacement rate in October 2020 and August - September 2021	0.6	n/a
Coronavirus Job Retention Scheme: maximum payment to furloughed workers in September 2020 and July 2021	1875#m	n/a
Self-Employment Income Support Scheme: default max earnings subsidy paid by the state (rate)	0.8	n/a
Self-Employment Income Support Scheme: default max earnings subsidy paid by the state (amount)	2500#m	n/a
Self-Employment Income Support Scheme: default max earnings subsidy paid by the state (rate) between August and October 2020	0.7	n/a
Self-Employment Income Support Scheme: default max earnings subsidy paid by the state (amount) between August and October 2020	2190#m	n/a
Coronavirus Job Retention Scheme earnings contribution paid by employer included in earnings and original income variables in UKMOD	Yes	n/a

Coronavirus Job Retention Scheme earnings subsidy paid by the state included in original income and replacement income in UKMOD	Yes	n/a
Self-Employment Income Support Scheme earnings subsidy paid by the state included in original income and replacement income in UKMOD	Yes	n/a
Coronavirus Job Retention Scheme earnings subsidy paid by the state included in non-means tested benefits in UKMOD	Yes	n/a
Self-Employment Income Support Scheme earnings subsidy paid by the state included in non-means tested benefits in UKMOD	Yes	n/a
National Insurance Contributions paid by the state for furloughed workers included in Credited National Insurance Contributions in UKMOD	Yes	n/a
Earnings subsidies from Coronavirus Job Retention Scheme and Self-Employment Income Support Scheme counted to income disregards for benefits	Yes	n/a
Local Housing Allowance (average rates by Government Office Region)		
North East England		
Shared accommodation	67.35#w	61.83#w
1 bed	83.84#w	82.25#w
2 bed	96.56#w	95.45#w
3 bed	113.72#w	109.49#w
4 bed	156.64#w	146.2#w
North West England and Merseyside		
Shared accommodation	68.81#w	61.37#w
1 bed	94.6#w	89.45#w
2 bed	114.28#w	108.67#w
3 bed	136.66#w	127.39#w
4 bed	182.85#w	165.68#w
Yorkshire and Humberside		
Shared accommodation	66.24#w	61.79#w
1 bed	94.1#w	86.86#w
2 bed	113.41#w	106.34#w
3 bed	130.53#w	122.12#w
4 bed	170.23#w	156.79#w
East Midlands		
Shared accommodation	72.31#w	62.02#w
1 bed	97.95#w	86.87#w
2 bed	122.01#w	108.12#w
3 bed	141.08#w	123.9#w
4 bed	181.96#w	161.1#w
West Midlands		
Shared accommodation	72.95#w	66.99#w

1 bed	109.41#w	99.26#w
2 bed	134.88#w	123.72#w
3 bed	159.83#w	143.42#w
4 bed	208.62#w	187.04#w
East of England		
Shared accommodation	78.64#w	69.16#w
1 bed	138.08#w	120.62#w
2 bed	171.52#w	150.94#w
3 bed	209.11#w	180.41#w
4 bed	267.82#w	233.73#w
London		
Shared accommodation	121.26#w	99.58#w
1 bed	250.83#w	224.45#w
2 bed	309.78#w	276.65#w
3 bed	378.49#w	332.88#w
4 bed	480.33#w	404.22#w
South East England		
Shared accommodation	84.92#w	75.89#w
1 bed	152.58#w	136.49#w
2 bed	191.51#w	171.74#w
3 bed	235.47#w	205.34#w
4 bed	310.47#w	278.29#w
South West England		
Shared accommodation	81.08#w	69.55#w
1 bed	117.52#w	106.84#w
2 bed	148.27#w	135.65#w
3 bed	179.62#w	162.62#w
4 bed	236#w	210#w
Wales		
Shared accommodation	64.34#w	56.81#w
1 bed	86.06#w	80.65#w
2 bed	107.24#w	100.56#w
3 bed	121.67#w	115.38#w
4 bed	156.78#w	145.24#w
Scotland		
Shared accommodation	71.49#w	63.88#w
1 bed	94.48#w	88.96#w
2 bed	120.38#w	111.39#w
3 bed	147.61#w	133.64#w
4 bed	225.66#w	188.11#w
Northern Ireland		
Shared accommodation	56.52#w	48.19#w
1 bed	80.61#w	74.16#w
2 bed	95.44#w	88.84#w
3 bed	105.76#w	98.88#w
4 bed	120.75#w	113.48#w

National Insurance Contributions paid by the state for furloughed workers…		
...if Coronavirus Job Retention Scheme subsidy received and survey interview between Apr-Jul...	... National Insurance Contributions paid by the state proportional to the CJRS subsidy as a % of total employee earnings	n/a
...if no Coronavirus Job Retention Scheme subsidy received or subsidy received and survey interview NOT between Apr-Jul...	...no National Insurance Contributions	n/a

Note:
Some policies introduced as response to the Covid-related economic crisis directly depend on the labour market activity status in SimPaths (Self-Employment Income Support Scheme; Coronavirus Job Retention Scheme). The removal of such policies occurs within SimPaths, not through modification of the UKMOD policy scenario. This requires the use of two branches of SimPaths (which are both available on the GitHub repository). Specifically, the baseline scenario uses SimPaths branch "development_UK-multirun-v2" and UKMOD policy scenario "uk_2020_std". The counterfactual scenario uses SimPaths branch "development_uk-multirun_v2_noCovidPolicies_v2" and UKMOD policy scenario "uk_2020precovid19_std". The policy scenario files are included in the A2.51+ version of UKMOD, which is publicly available without registration from https://www.microsimulation.ac.uk/ukmod/access/ .
